# Impact of low blood culture usage on rates of antimicrobial resistance

**DOI:** 10.1016/j.jinf.2020.10.040

**Published:** 2021-03

**Authors:** Cherry Lim, Viriya Hantrakun, Nittaya Teerawattanasook, Pramot Srisamang, Prapit Teparrukkul, Nithima Sumpradit, Paul Turner, Nicholas PJ Day, Ben S Cooper, Sharon J Peacock, Direk Limmathurotsakul

**Affiliations:** a Mahidol Oxford Tropical Medicine Research Unit, Faculty of Tropical Medicine, Mahidol University, Bangkok, Thailand; b Centre for Tropical Medicine and Global Health, Nuffield Department of Medicine, University of Oxford, Oxford, United Kingdom; c Department of Clinical Pathology, Sunpasitthiprasong Hospital, Ubon Ratchathani, Thailand; d Pediatrics, Sunpasitthiprasong Hospital, Ubon Ratchathani, Thailand; e Department of Internal Medicine, Sunpasitthiprasong Hospital, Ubon Ratchathani, Thailand; f Thai Food and Drug Administration, Ministry of Public Health, Bangkok, Thailand; g Cambodia Oxford Medical Research Unit, Angkor Hospital for Children, Siem Reap, Cambodia; h University of Cambridge, Addenbrooke's Hospital, Cambridge, United Kingdom; i Department of Tropical Hygiene, Faculty of Tropical Medicine, Mahidol University, Bangkok, Thailand

**Keywords:** Antimicrobial resistant, Surveillance, Blood culture, Proportion, Incidence, Rate

## Abstract

**Objectives:**

The magnitude of impact caused by low blood culture utilization on estimates of the proportions and incidence rates of antimicrobial-resistant (AMR) bacterial infections is largely unknown.

**Methods:**

We used routine electronic databases of microbiology, hospital admission and drug prescription at Sunpasitthiprasong Hospital, Ubon Ratchathani, Thailand, from 2011 to 2015, and bootstrap simulations.

**Results:**

The proportions of *Escherichia coli* and *Klebsiella pneumoniae* bacteraemias caused by 3rd generation cephalosporin resistant isolates (3GCREC and 3GCRKP) were estimated to increase by 13 and 24 percentage points (from 44% to 57% and from 51% to 75%), respectively, if blood culture utilization rate was reduced from 82 to 26 blood culture specimens per 1,000 patient-days. Among patients with hospital-origin bloodstream infections, the proportion of 3GCREC and 3GCRKP whose first positive blood culture was taken within ±1 calendar day of the start of a parenteral antibiotic at the study hospital was substantially lower than those whose first positive blood culture was taken later into parenteral antibiotic treatment (30% versus 79%, *p*<0.001; and 37% versus 86%, *p*<0.001). Similar effects were observed for methicillin-resistant *Staphylococcus aureus*, carbapenem-resistant *Acinetobacter* spp. and carbapenem-resistant *Pseudomonas aeruginosa*.

**Conclusion:**

Impacts of low blood culture utilization rate on the estimated proportions and incidence rates of AMR infections could be high. We recommend that AMR surveillance reports should additionally include blood culture utilization rate and stratification by exposure to a parenteral antibiotic at the hospital.

## Introduction

Antimicrobial-resistance (AMR) surveillance reports are commonly used to monitor trends, inform recommendations for empirical therapy, estimate the burden of AMR, and assess the impact of local, national and global interventions.^1^, ^2^, ^3^ Such reports can be generated from collated bacterial culture results of priority specimens, particularly from blood, that have been taken for clinical purposes.^1^^,^^4^ The reports are also commonly referred to as cumulative antibiogram reports and cumulative antimicrobial susceptibility reports.^5^ In 2015, the World Health Organization (WHO) launched the Global Antimicrobial Resistance Surveillance System (GLASS), promoting the use of globally agreed and standardized methods for compiling and reporting data locally and nationally.^1^ The recommended standardization includes how data should be de-duplicated and how to use specimen collection and hospital admission dates to classify the origin of infection into community- or hospital-origin as a proxy for community- or hospital-acquired infection, respectively. Estimated parameters include the proportions of patients with bloodstream infection (BSI) caused by AMR isolates (using an isolate-based surveillance approach), and incidence rates of patients with bloodstream infection (BSI) caused by AMR isolates in the tested population (using a sample-based surveillance approach) stratified by origin of infection (community or hospital).^1^

It is well recognised that a low blood culture utilization rate can bias AMR surveillance data,^1^^,^^4^^,^^6^ but the magnitude of impact from this on estimates of proportions and incidence rates of AMR infections is largely unknown.^7^ In hospitals in low and middle-income countries (LMICs), patients with severe infectious diseases are frequently treated empirically and blood culture is frequently sampled after empirical treatment failure. Here, we quantify the magnitude of effect caused by low blood culture utilization rates in LMICs on estimates of proportions and incidence rates of AMR infections. We also develop and evaluate a new parameter to represent blood culture utilization in LMICs.

## Materials and methods

### Study design

We conducted a simulation study using the routine electronic databases of microbiology, hospital admission and drug prescription at Sunpasitthiprasong Hospital, Ubon Ratchathani, Thailand, from 2011 to 2015. Ethical permission for this study was obtained from the Oxford Tropical Research Ethics Committee (Ref. 557-17), and the ethical committee of Sunpasitthiprasong Hospital (Ref. 005/2560). The committees waived the requirement to obtain individual informed consent due to the study design and minimal risk to the subjects.

### Definitions

We used the definitions of infection origin as proposed by WHO GLASS.^1^ In brief, community-origin (or hospital-origin) BSI was defined for patients in the hospital within (or longer than) the first two calendar days of admission when the first blood specimens culture positive for a pathogen were taken.^1^ A blood culture episode was defined as all blood culture specimens taken within two calendar days beginning when a blood culture specimen was taken.

We focused on rates of blood culture utilization in relation to BSI caused by *Escherichia coli, Klebsiella pneumoniae, Staphylococcus aureus, Acinetobacter spp.* and *Pseudomonas aeruginosa* because these are the top five pathogens attributable to deaths caused by AMR infections in Thailand^8^ and the EU and the European Economic Area.^9^

Proportions of 3rd generation cephalosporin resistant *E. coli* (3GCREC) and *K. pneumoniae* (3GCRKP), methicillin-resistant *S. aureus* (MRSA), and carbapenem-resistant *Acinetobacter* spp. (CRACI) and *P. aeruginosa* (CRPA) were defined as the ratio of the number of patients having a blood culture positive for 3GCREC, 3GCRKP, MRSA, CRACI and CRPA, and the total number of patients with at least one positive blood culture with the given organism, respectively. Only the first isolate per patient, per pathogen, per study period was included in the analyses. Supplementary text describes how all parameters were estimated.^1^^,^^10^

We developed a new variable ‘proportion of patients having a blood culture taken within ±1 calendar day of the day when a parenteral antibiotic was started and continued for at least four consecutive days.’ We included patients who died, were discharged to a hospice or transferred to another hospital before completing four consecutive days of antibiotics and had antibiotics continuously until the day prior to death, a hospice discharge or transfer, respectively.^11^ We included consecutive calendar days with any parenteral antibiotics after a parenteral antibiotic was started. We used four consecutive days of parenteral antibiotics as a proxy for presumed severe infection,^11^ in which blood culture is generally recommended.^12^^,^^13^

### Statistical analysis

We used bootstrap resampling with 200 iterations for each simulation. The median and the 2.5th and 97.5th percentiles are presented. To quantify the potential magnitude of impact caused by low blood culture utilization rate in LMICs, we simulated datasets in which only 50%, 25% and 10% of all first blood culture episodes were included using data from the patients with blood cultures and bootstrap simulations. We included in the analyses our data from all repeated blood culture episodes to represent delayed blood culture if the first blood culture episodes were not sampled. Proportions were compared between groups using Chi-square test or Fisher's Exact test. Continuous variables were compared between groups using Kruskal–Wallis test. All analyses were performed using STATA 14.2 (StataCorp LP, College Station, Texas).

## Results

### Baseline characteristics

**Fig. 1.**
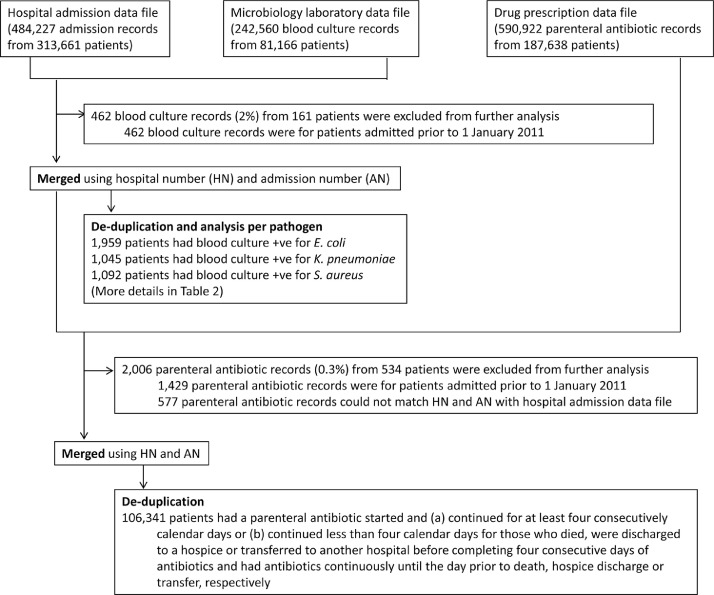
Study data flow diagram. * A blood culture episode was defined as all blood culture specimens collected within two calendar days beginning when a blood culture specimen was collected.

Of 313,661 patients admitted to the study hospital from 2011 to 2015, 81,036 patients had at least one blood culture taken (total 242,098 blood cultures) (Table 1 and Fig. 1). Total patient days were 2,956,643, giving a blood culture utilization rate of 82 blood cultures per 1,000 patient-days. Around three quarters of patients (62,756 of 81,036 cases, 77%) had a single blood culture episode during their admission (defined as all blood cultures collected within two calendar days from the first specimen). 11,410 (14%), 3,751 (5%), 1,538 (2%) and 1,581 (2%) patients had two, three, four and at least five blood culture episodes during their admissions, respectively. Among patients who had repeated blood culture episodes, the median time between the first and second blood culture episode was 5 calendar days (IQR, 3–9 days).

**Table 1.** Baseline characteristics of the study population in Sunpasitthiprasong Hospital.

General parameters for the hospital and from hospital admission data file	Sunpasitthiprasong Hospital, Thailand from 2011 to 2015 (5 years)
Hospital bed capacity (beds)	1,183 beds (in 2015)
Total number of inhabitants in the catchment area	1,844,669 population (in 2015)
Total number of admission records	484,227 admissions
Total number of admission records with in-hospital mortality outcome	15,589 admissions
Total number of patient-days (up to 31Dec2015)	2,956,643 patient-days
Total number of inpatients (de-duplicated patients)	313,661 patients
*General parameters from microbiology laboratory data file*	
Number of blood culture specimens	242,098 samples
Number of blood culture results recorded as “no growth”	216,558 samples (89%)
Number of patients sampled for blood culture (de-duplicated patients)	81,036 patients
*General parameters from drug prescription data file*	
Number of parenteral antibiotic records	590,922 records
Number of patient-days with a parenteral antibiotic (de-duplicated patient-days)*	1,456,027 patient-days
Number of patients prescribed with a parenteral antibiotic (de-duplicated patients)	187,302 patients

⁎ If a patient had more than one parenteral antibiotic on a given day, the number was counted as one patient-day with a parenteral antibiotic.

We identified 1,959, 1,045, 1,092, 1,000 and 450 patients with blood cultures positive for *E. coli, K. pneumoniae, S. aureus, Acinetobacter* spp. and *P. aeruginosa*, respectively. The proportions of 3GCREC, 3GCRKP, MRSA, CRACI and CRPA were 44%, 51%, 23%, 65% and 28% respectively (Table 2). The proportions of 3GCREC, 3GCRKP, MRSA, CRACI and CRPA in BSI of hospital-origin were significantly higher than those in BSI of community-origin (all *p*<0.001, Table 2). Estimated incidence rates of 3GCREC, 3GCRKP, MRSA, CRACI and CRPA BSI per 100,000 population per year were 9.5, 5.8, 2.7, 7.0 and 1.4, respectively (Tables S1–S5). Using a sample-based approach proposed by WHO GLASS, we estimated incidence rates for 3GCREC, 3GCRKP MRSA, CRACI and CRPA BSI per 100,000 tested patients of 1,071, 659, 311, 798 and 154, respectively.

**Table 2.** Proportions of patients with blood cultures positive for antibiotic-resistant isolates for five bacterial species.

Species	Resistance of interest	Proportion with specified resistance			*P* value
		Total	BSI of community-origin*	BSI of hospital-origin*	
*Escherichia coli*	Third-generation cephalosporin resistance (3GCREC)	44% (868/1,959 patients)	42% (631/1,514 patients)	53% (237/445 patients)	<0.001
*Klebsiella pneumoniae*	Third-generation cephalosporin resistance (3GCRKP)	51% (534/1,045 patients)	34% (193/569 patients)	72% (341/476 patients)	<0.001
*Staphylococcus aureus*	Methicillin resistance (MRSA)	23% (252/1,092 patients)	14% (103/750 patients)	44% (149/342 patients)	<0.001
*Acinetobacter* spp	Carbapenem resistance (CRACI)	65% (647/1,000 patients)	33% (80/242 patients)	75% (567/758 patients)	<0.001
*Pseudomonas aeruginosa*	Carbapenem resistance (CRPA)	28% (125/450 patients)	14% (23/170 patients)	36% (102/280 patients)	<0.001

⁎ Blood stream infection (BSI) of community-origin was defined as patients in hospital for ≤2 calendar days when the first blood-culture positive for the pathogen was taken, and BSI of hospital-origin was defined as patients admitted for >2 calendar days when the first blood-culture positive for pathogen was taken ^1^. Only the first isolate per patient, per pathogen, per study period was included in the analysis.

187,302 patients (60%) received at least one parenteral antibiotic (Fig. S1). The most common agents in use being ceftriaxone (346,167 patient-days), carbapenem drugs (253,466 patient-days) and ceftazidime (251,548 patient-days). The proportion of patients who had a blood culture taken within ±1 calendar day of the start of parenteral antibiotic treatment that was then continued for at least four consecutive days was 44% (47,132/106,341). This rate was higher in patients who began a parenteral antibiotic within the first two calendar days of hospital admission (46%; 41,489/90,685) compared with after two calendar days (36%; 5643/15,656; *p*<0.001). This rate was also higher in patients first admitted to internal medicine or pediatric wards (80%; 38,820/48,579) versus other wards including surgery and orthopedics (14%; 8312/57,762, *p*<0.001).

### Impact of incorrect blood culture practice on estimated proportions of AMR infections

**Fig. 2.**
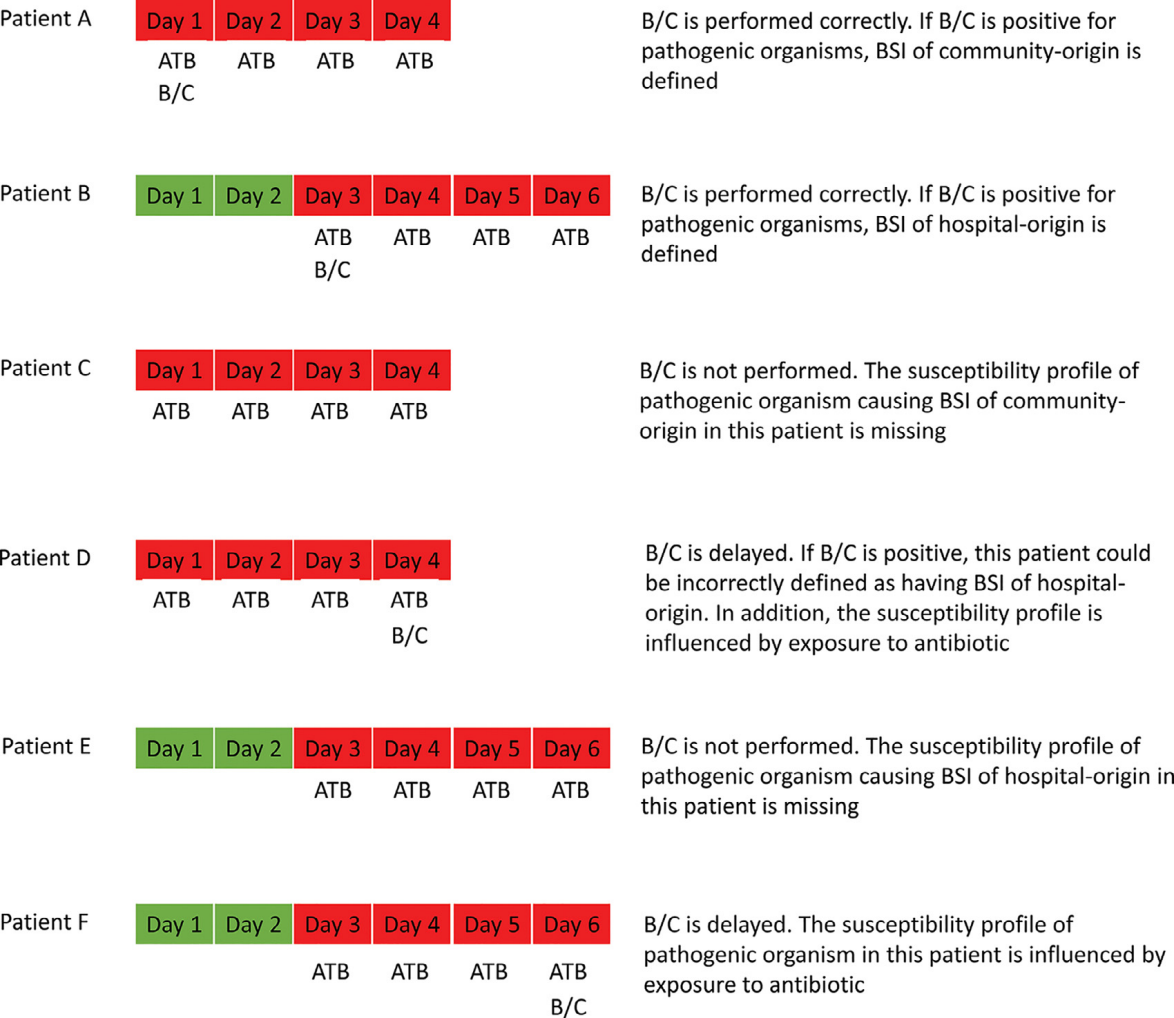
Diagrams illustrating how AMR surveillance data could be influenced if blood culture is delayed or not performed. Green and red blocks indicate the non-infectious and infectious patient state, respectively. B/C and ATB indicate blood culture collection date and the date receiving a parenteral antibiotic, respectively. Patients A and B have BSI of community-origin and hospital-origin, respectively, which could be detected through appropriate blood culture sampling within ±1 calendar day of the start of a parenteral antibiotic. Patients C and D have BSI of community-origin who are either not cultured at all and so data points will be missing (C), or have delayed culture that results in errors caused by exposure to ATB and incorrect assignment of a positive culture result as BSI of hospital-origin (D). Patients E and F have BSI of hospital-origin who are either not cultured at all and so data points will be missing (E), or have a delayed culture that results in errors caused by exposure to ATB (F).

Estimates of the proportions of AMR infection in a given population could be influenced by variation in blood culture practice, specifically when samples are not systematically taken in relation to the time of presentation with features of infection and commencement of antibiotic treatment. Fig. 2 provides a descriptive framework based on six possible scenarios where patients are either correctly (cases A & B) or incorrectly sampled (cases C to F), together with the effect of incorrect sampling on data generated for the proportions of AMR infection.

**Fig. 3.**
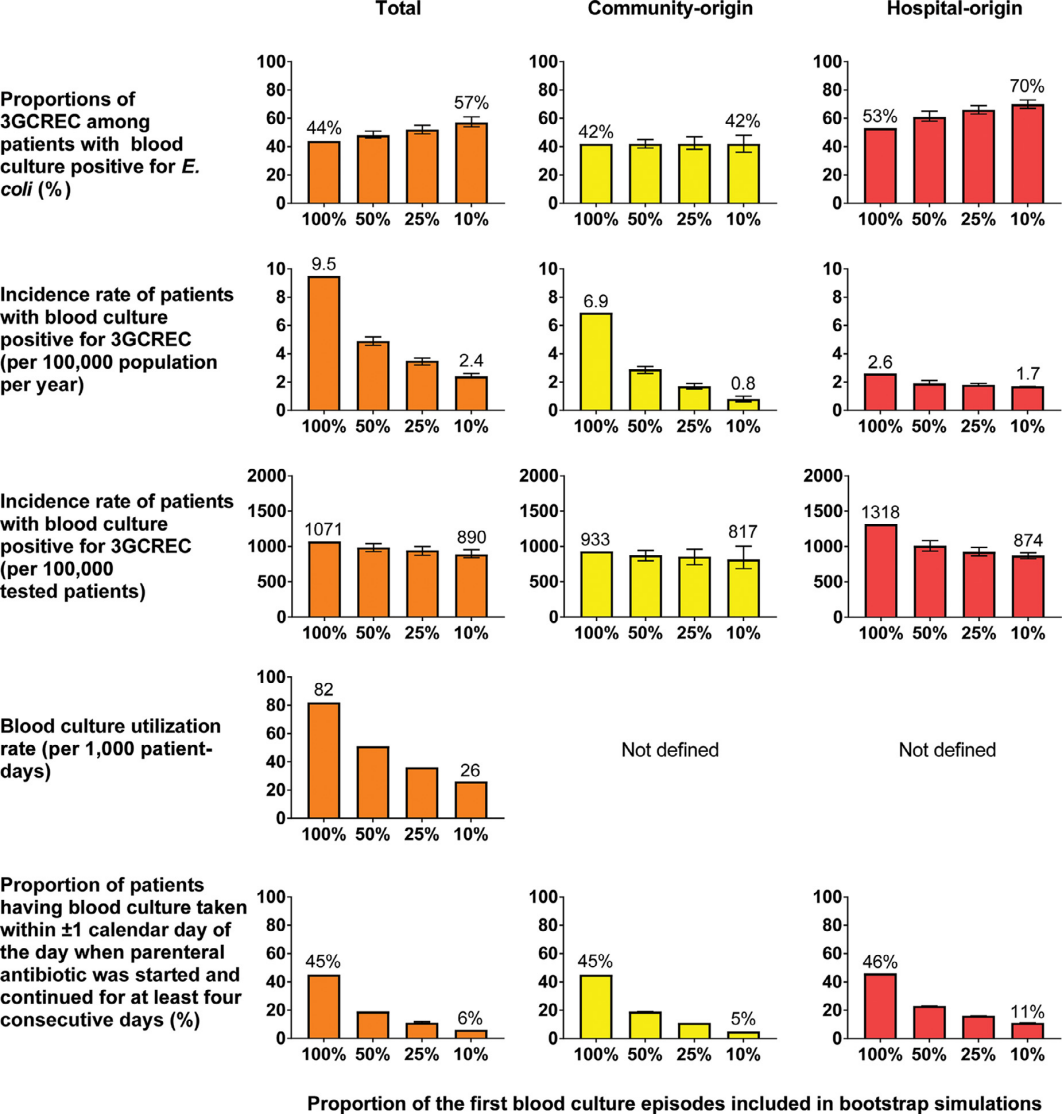
Estimated proportions and incidence rates of 3rd generation cephalosporin resistant *E. coli* (3GCREC) if 50%, 25% and 10% of the first blood culture episodes* were sampled. * A blood culture episode was defined as all blood culture specimens collected within two calendar days beginning when a blood culture specimen was collected.

In bootstrap simulations, the proportion of 3GCREC was estimated to increase by 13 percentage points (from 44% to 57%) if only 10% of the first blood culture episodes were sampled (Fig. 3, row 1). The most probable proportion of 3GCREC in patients with BSI of community-origin (42%) would not change, but the proportion could range from 36% to 48% due to lower sample sizes as shown by its 95% bootstrap confidence intervals (BCI 36–48%). The estimated proportion of 3GCREC in patients with BSI of hospital-origin would increase by 17 percentage points (from 53% to 70%). Similar effects were observed for 3GCRKP*,* MRSA, CRACI and CRPA (Figs. S2–S5).

### Impact of incorrect blood culture practice on estimated AMR rates

The rate of AMR infections per 100,000 population per year is commonly used to estimate and monitor burden of AMR infections,^2^^,^^3^ and was estimated in our simulations. The incidence rate of patients with 3GCREC BSI per 100,000 population per year was estimated to decrease by 75% (from 9.5 to 2.4; Fig. 3, row 2) if 10% of the first blood culture episode were sampled. This was because the number of patients with 3GCREC BSI identified (numerator for the incidence rate) would decrease from 868 cases to 224 (95% BCI 211–241; Table S1), while the total population coverage (denominator for the incidence rate) was constant. The decrease in the estimated incidence rate of community-origin 3GCREC per 100,000 population (from 6.9 to 0.8) was considerably higher than that for hospital-origin 3GCREC (from 2.6 to 1.7). Similar effects were observed for 3GCRKP*,* MRSA, CRACI and CRPA (Figs. S2–S5).

The incidence rate of 3GCREC BSI per 100,000 tested patients was estimated to decrease by 17% (from 1,071 to 890; Fig. 3, row 3) if 10% of the first blood culture episodes were sampled. However, the incidence rate of 3GCRKP, MRSA, CRACI and CRPA BSI per 100,000 tested patients were estimated to increase by 71% (from 659 to 1,128), 108% (from 311 to 648), 147% (from 798 to 1,975) and 132% (154 to 358) respectively (Tables S2–S5).

### Impact of incorrect blood culture practice on parameters representing blood culture utilization

Blood culture utilization rate per 1,000 patient-days is commonly used to represent the practice of blood culture sampling and was estimated in our simulations. The utilization rate was estimated to decrease from 82 to 26 per 1,000 patient days if 10% of the first blood culture episodes were sampled (Fig. 3 row 4). However, this parameter could not be stratified by origin of infection.

The proportion of patients having a blood culture taken within ±1 calendar day of the day when a parenteral antibiotic was started and continued for at least four consecutive days would also correspondingly reduce from 44% to 6% if 10% of the first blood culture episodes were sampled (Fig. 3, row 5). The parameter could be stratified by origin of infection. In our simulations, the decline was greater among patients at risk of BSI of community-origin (from 46% to 5%) than that of hospital-origin (from 36% to 11%).

### Proportions of AMR infections stratified by exposure to a parenteral antibiotic

**Fig. 4.**
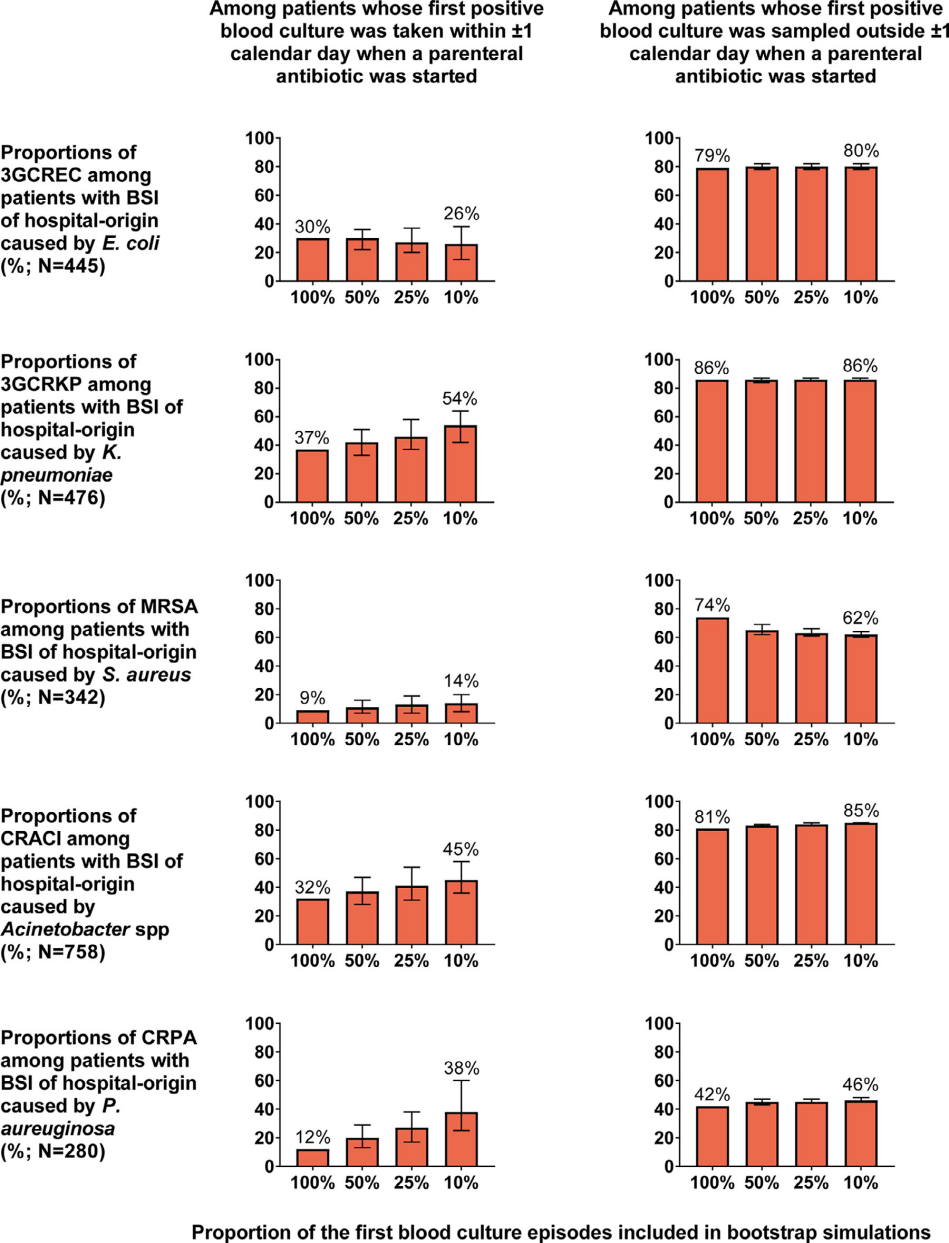
Proportions of 3GCEC, 3GCRKP, MRSA, CRACI and CRPA in patients with BSI of hospital-origin stratified by exposure to a parenteral antibiotic at the study hospital and if 50%, 25% and 10% of the first blood culture episodes* were sampled. * A blood culture episode was defined as all blood culture specimens collected within two calendar days beginning when a blood culture specimen was collected. Characteristics of patients stratified by exposure to a parenteral antibiotic are presented in Tables S6–S10.

We stratified patients with BSI of hospital-origin by exposure to a parenteral antibiotic at the study hospital (Fig. 4). The proportion of 3GCREC in patients whose first positive blood culture was taken within ±1 calendar day of the start of a parenteral antibiotic was substantially lower compared with patients whose first positive blood culture was taken later into parenteral antibiotic treatment (30% versus 79%, *p*<0.001). The median duration of hospital admission in the former group was around half that of the latter group (5 vs. 10.5 days, *p*<0.001, Table S6). The median duration of exposure to a parenteral antibiotic at the study hospital in the former group was significantly lower than that of the latter group was (0 vs. 7.5 days, *p*<0.001, Table S6). Similar effects were observed for 3GCRKP, MRSA, CRACI and CRPA (Fig. 4).

We also explored whether (a) the large difference between the proportion of AMR infections from blood cultures taken within ±1 calendar day of the start of a parenteral antibiotic versus the proportion of AMR infections from blood cultures taken later, and (b) the low proportion of AMR infections from blood cultures taken within ±1 calendar day of the start of a parenteral antibiotic would still be observed when blood culture was often delayed. We found that the large difference between the proportion of 3GCREC from blood cultures taken within ±1 calendar day of the start of a parenteral antibiotic versus the proportion of 3GCREC from blood cultures taken later was estimated to be present even if 10% of the first blood culture episodes were sampled. The most probable proportion of 3GCREC from blood culture taken ±1 calendar day of the start of a parenteral antibiotic was estimated to decrease by 4 percentage point (from 30% to 26%; Fig. 4 row 1).

The large difference between proportions of 3GCRKP, MRSA and CRACI from blood cultures taken within ±1 calendar day of the start of a parenteral antibiotic versus the corresponding proportions from blood cultures taken later was also estimated to be present if 10% of the first blood culture episodes were sampled (Fig. 4). The most probable proportions of 3GCRKP, MRSA, CRACI and CRPA in patients whose first positive blood culture was taken within ±1 calendar day of the start of a parenteral antibiotic were estimated to increase by 17 percentage points (from 37 to 54%), 5 percentage points (from 9 to 14%), 13 percentage points (32 to 45%) and 26 percentage points (from 12 to 38%), if 10% of the first blood culture episodes were sampled (Tables S7–S10).

## Discussion

We illustrate that estimates of the proportions and incidence rates of AMR infections could be considerably changed due to low blood culture utilization rates in LMIC settings, where patients are frequently treated empirically and blood culture is frequently sampled after empirical treatment failure. This could occur even if there are no changes in true susceptibility profiles of pathogenic organisms and in true infection rates in that environment. We show that the proportions of 3GCREC, 3GCRKP, MRSA, CRACI and CRPA could rise by 13, 24, 20, 16 and 15 percentage points if blood culture utilization rate was reduced from 82 to 26 per 1,000 patient days. The incidence rates of AMR infections per 100,000 population per year could fall considerably. However, the changes in incidence rates of AMR infections per 100,000 tested patients could vary by organism, ranging from a 17% decrease for 3GCREC to a 147% increase for CRACI. We also show that the proportions of AMR isolates from blood sampled within ±1 calendar day were significantly lower than those from blood sampled outside ±1 calendar day when a parenteral antibiotic was started at the study hospital.

Multiple reasons could contribute to the increase of total proportions of AMR infections if blood culture is frequently delayed. First, the decrease in total numbers of patients with BSI of community-origin is more than the decrease in those with BSI of hospital-origin (Tables S1–S5). The former has lower proportions of AMR infections compared to the latter and so the total proportions of AMR infections increase. Second, the proportions of AMR infections in patients with BSI of hospital-origin increase. This could be because longer hospital stays and prior exposure to parenteral antibiotics can increase the risk of AMR infections,^14^^,^^15^ profiles of pathogenic organisms from patients with BSI of hospital-origin who improve after empirical treatment are missing (the scenario of Patient E in Fig. 2), and prior exposure to parenteral antibiotics can also reduce an opportunity to detect non-AMR bacteria from patients with non-AMR infections^16^ (the scenario of Patient F in Fig. 2).

Blood culture utilization rate and the new parameter (the proportion of patients having a blood culture taken within ±1 calendar day of the day when a parenteral antibiotic was started at the study hospital and continued for at least four consecutive days) performed well in our simulations. They correlated well with the reduction of blood culture sampling in bootstrap simulations. The new parameter is also useful to understand blood-culturing practices in different settings (e.g. with different origin of infection and in different wards).

High blood culture utilization rate in Thailand, a middle-income country, is consistent with our previous finding that the total number of blood culture bottles used at the study hospital had been rising from 5,235 bottles per year in 1995 to 56,719 bottles per year in 2015.^17^ A high proportion of patients having blood culture sampled within ±1 calendar day of the day when a parenteral antibiotic was started (80%) in internal medicine and pediatric wards is supported by a previous prospective observational study.^18^ In routine care at the study hospital, 84% of adult patients presenting with sepsis in internal medicine wards had a blood culture specimen collected and 89% received parenteral antibiotics within the first two calendar day of hospital admission.^18^ Blood culture utilization rate at the study hospital (82 blood culture specimens per 1,000 patient-days) is within the range of the number of blood culture sets tested per 1,000 patient-days reported in the Europe ranging from 6.7 in Latvia to 86.5 in France.^9^

## Limitations

Our study has several limitations. First, the magnitude of impact shown may not be generalizable to other settings. Second, the definition of origin of infection is only a proxy for community-acquired and hospital-acquired infection.^1^ A proportion of patients with BSI of community-origin could be caused by nosocomial infections acquired at transferring hospitals.^19^ Third, our data could not define whether two or more blood cultures obtained on the same day were drawn from a single phlebotomy (in which those specimens could be defined as a single blood culture set).^9^^,^^20^^,^^21^ Therefore, we reported blood cultures per 1000 patient-days, which is probably higher than the value of blood culture sets per 1000 patient-days.^9^^,^^20^ Fourth, we could not determine the impact caused by exposure to a parenteral antibiotic prior to hospital admission. Fifth, we could not determine why the proportion of patients having a blood culture taken within ±1 calendar day of the day when a parenteral antibiotic was started is low in the wards other than pediatric and internal medicine wards. This could be due to high proportion of contaminated and dirty procedures (e.g. open, fresh, accidental wounds), continuing antimicrobial prophylaxis after clean and clean-contaminated procedures (which is currently not recommended even in the presence of a drain),^22^^,^^23^ or other unknown causes.

## Recommendations

Based on our findings, we proposed a set of recommendations. First, we support the recommendations that AMR surveillance reports should present all parameters with stratification by origin of infection,^1^ and with clear terminology, methodology, and numbers of numerators and denominators used to calculate each parameter.^6^^,^^24^ This would allow people to compare and monitor proportions and incidence rates of AMR infections from different sources or settings, by being aware of or taking account of potential impact of different definitions and methods in the future.^6^^,^^24^

Second, AMR surveillance reports should report parameters representing blood culture utilization (such as blood culture utilization rates^9^^,^^20^ and the proportion of patients having a blood culture taken within ±1 calendar day of the day when a parenteral antibiotic was started at the study hospital and continued for at least four consecutive days). This is because the impact caused by low blood culture utilization could be high.

Third, incidence rates per 100,000 tested patients should be estimated and reported, especially in LMICs where blood culture utilization rate is low or unknown.^17^^,^^25^^,^^26^ This is because the magnitude of impact on the incidence rates per 100,000 tested patients is likely to be lower than that on the incidence rate per 100,000 population per year if blood culture utilization rate is low (Fig. 3). The incidence rates per 100,000 population per year is still a good parameter for monitoring total AMR burden in high-income countries where blood culture utilization rates are considered high.^2^^,^^9^

Fourth, interpreting AMR trend needs to take account of the change of blood culture utilization rate. This is because if a hospital improves their blood culture utilization rate over time (e.g. doubling their utilization rate), observed proportions of AMR infections could considerably decline while observed incidence rates of AMR infections (per 100,000 population per year) could considerably increase even if there are no changes in true susceptibility profiles of pathogenic organisms and in true infection rates in that environment over time.

Fifth, hospitals in LMICs with a low blood culture utilization rate should use AMR surveillance reports stratified by exposure to an empirical antibiotic at the study hospital to guide choice of first-line empiric antimicrobial therapy rather than the total antibiogram. This is because the cumulative antibiogram reports mainly based on blood culture after failure of the first empiric treatment should not be used to guide choice of the first empiric therapy. This is similar to the recommendations for tuberculosis, where proportions of multi-drug resistant tuberculosis are stratified for new (never treated) tuberculosis cases and previously treated tuberculosis cases, and the choice of first-line anti-tuberculosis drugs is based on the proportion of drug-resistant tuberculosis found in new cases.^27^, ^28^, ^29^

## Funding

The study is supported by Ministry of Public Health Thailand and Wellcome Trust of Great Britain (106698/Z/14/Z). C.L. and D.L. were supported by a Training Fellowship (grant number 206736/Z/17/Z) and an Intermediate Fellowship (grant number 101103/Z/13/Z), respectively, from the Wellcome Trust. B.S.C. was supported by The Medical Research Council and 10.13039/501100000278Department for International Development (grant number MR/K006924/1).

## Transparency declarations

We declare that no competing interests exist.

## Declaration of Competing Interest

None.

## Appendix Supplementary materials

Image, application 1
