# Supplementary material for: Impact of low blood culture usage on rates of antimicrobial resistance
Source: J Infect. 2021 Mar;82(3):355–62. doi: 10.1016/j.jinf.2020.10.040 (PMC7994019; doi:10.1016/j.jinf.2020.10.040)
Supplement: Supplementary file 1 [file mmc1.docx]

**Supplementary text**

**Supplementary methods**

**Study Population**

Ubon Ratchathani province is the second largest province in northeast Thailand (Table 1). Sunpasitthiprasong Hospital is the referral center for all district hospitals in the province and provincial hospitals in the adjacent provinces. The hospital is equipped with a microbiology laboratory that provides bacterial culture services, while most district hospitals do not have microbiology facilities. From 2011 to 2015, the BD BACTEC automated blood culture system (Becton-Dickinson, Sparks, MD, USA) was used at the study hospital. All bacteria isolated were identified using conventional bacterial identification methods.^1^ Antimicrobial susceptibility was determined using the disc diffusion method according to Clinical and Laboratory Standards Institute (CLSI) guidelines.^1-3^

**Data collection**

The microbiology data obtained included patient hospital number, admission number, specimen collection date, specimen type, culture result and antibiotic susceptibility testing result. Hospital admission data were from routine in-patient discharge reports including patient hospital number, admission number, gender, age, admission date, discharge date and in-hospital discharge outcome. Drug prescription data obtained included hospital number, admission number, drug names, administration routes, start date and stop date. All data were obtained as electronic files in a readily accessible format.

**Simulation**

To quantify the potential magnitude of error, we simulated datasets in which only 50%, 25% and 10% of all first blood culture episodes were included using data from the patients with blood cultures. We included in this analysis our data from all repeated blood culture episodes to represent delayed blood culture if the first blood culture episodes were not sampled. We then explored how the parameter ‘Incidence rates of new cases of AMR infections per 100,000 tested patients’ proposed by WHO GLASS would change if blood culture utilization rate was lowered. Having observed that the proportions of AMR infections in patients with BSI of hospital-origin were higher when blood culture was delayed, we explored whether this was associated with antibiotic exposure. We stratified patients with BSI of hospital-origin by exposure to a parenteral antibiotic.

**Parameters for AMR infections were calculated as follows:**

| Proportion of patients with blood culture positive for resistant strains (%) | | = | | Number of patients with blood culture positive for resistant strains of bacterial species  (per pathogen, antibiotic and origin of infection) | |
| --- | --- | --- | --- | --- | --- |
|  |  |  |  | Total number of patients with blood culture positive for bacterial species during the reporting period  (per pathogen and origin of infection) | |
|  | |  | |  | |
|  |  |  |  |  | |
| Incidence rates of patients with bloodstream infection  (per 100,000 population per year) | | = | | Number of new patients with blood culture positive for bacterial species during reporting period * 100,000  (per pathogen, antibiotic and origin of infection) | |
|  |  |  |  | Total financial year population during reporting period | |
|  | |  | |  | |
|  |  |  |  |  | |
| Incidence rates of patients with bloodstream infection  (per 100,000 tested patients) | | = | | Number of new patients with blood culture positive for bacterial species during reporting period * 100,000  (per pathogen and antibiotic) | |
|  |  |  |  | Total number of patients with blood culture performed  during the reporting period | |
| Incidence rates of patients with bloodstream infection of community-origin  (per 100,000 tested patients) | | = | | Number of new patients with the first blood culture positive for bacterial species collected within the first two calendar days of admissions * 100,000  (per pathogen and antibiotic) | |
|  |  |  |  | Total number of patients with the first blood culture performed within the first two calendar days of admissions during the reporting period | |
|  | |  | |  | |
|  |  |  |  |  | |
| Incidence rates of patients with bloodstream infection of hospital-origin  (per 100,000 tested patients) | | = | | Number of new patients with the first blood culture positive for bacterial species collected after the first two calendar days of admissions  and the first blood culture performed after the first two calendar days of admissions * 100,000  (per pathogen and antibiotic) | |
|  |  |  |  | Total number of patients with the first blood culture performed after the first two calendar days of  admissions during the reporting period | |
|  | |  | |  | |
|  |  |  |  |  | |

**Parameters for culturing practices were calculated as follow:**

| Blood culture utilization rate  (per 1,000 patient-days) | = | Number of blood cultures used during reporting period * 1,000 |
| --- | --- | --- |
|  |  | Total patient-days occupancy during reporting period |
|  |  |  |
|  |  |  |
| Proportion of patients having blood culture sampled within ±1 calendar day of the day when a parenteral antibiotic was started and continued for at least four consecutive days  (new parameter generated for this study) | = | Total number of patients having blood culture sampled within ±1 calendar day when parenteral antibiotics was first prescribed and (a) continued for at least four consecutive days or (b) continued for less than four calendar days if the patients died, were discharged to a hospice or transferred to another hospital less than four days after antibiotics were started, and antibiotics were continued until the day prior to death, hospice discharge or transfer |
|  |  | Total number of patients who had parenteral antibiotics first prescribed and (a) continued for at least four consecutive days or (b) continued for less than four calendar days if the patients died, was discharged to hospice or transferred to another hospital less than four days after antibiotics were started, and antibiotics were continued until the day prior to death, hospice discharge or transfer |
|  |  |  |
|  |  |  |
| Proportion of patients at risk of bloodstream infection of community-origin and having blood culture sampled within ±1 calendar day of the day when a parenteral antibiotic was started and continued for at least four consecutive days  (new parameter generated for this study) | = | Total number of patients having blood culture sampled within ±1 calendar day of the day when a parenteral antibiotic was started within the first two calendar days of admission and (a) continued for at least four consecutive days or (b) continued for less than four calendar days if the patients died, were discharged to a hospice or transferred to another hospital less than four days after antibiotics were started, and antibiotics were continued until the day prior to death, hospice discharge or transfer |
|  |  | Total number of patients who had a parenteral antibiotic first started within the first two calendar days of admission and (a) continued for at least four consecutive days or (b) continued for less than four calendar days if the patients died, were discharged to a hospice or transferred to another hospital less than four days after antibiotics were started, and antibiotics were continued until the day prior to death, hospice discharge or transfer |
|  |  |  |
|  |  |  |
| Proportion of patients at risk of bloodstream infection of hospital-origin and having blood culture sampled within ±1 calendar day of the day when a parenteral antibiotic was started and continued for at least four consecutive days  (new parameter generated for this study) | = | Total number of patients having blood culture sampled within ±1 calendar day of the day when a parenteral antibiotic was started after the first two calendar days of admission and (a) continued for at least four consecutive days or (b) continued for less than four calendar days if the patients died, were discharged to a hospice or transferred to another hospital less than four days after antibiotics were started, and antibiotics were continued until the day prior to death, hospice discharge or transfer |
|  |  | Total number of patients who had a parenteral antibiotic started after the first two calendar days of admission and (a) continued for at least four consecutive days or (b) continued for less than four calendar days if the patients died, were discharged to a hospice or transferred to another hospital less than four days after antibiotics were started, and antibiotics were continued until the day prior to death, hospice discharge or transfer |

**Supplementary results**

**Baseline Characteristics**

A total of 15,589 patients had a discharge record of ‘in-hospital mortality’, giving crude fatality rate of 5% (15,589/313,661) among in-hospital patients during the study period. Of 484,227 admissions, 13,171 (3%) (from 13,016 patients) had a discharge record of ‘against advice and not improved’ and was assumed to be discharged to a hospice, and 22,506 (5%) (from 20,842 patients) had a discharge record of ‘transferred to another hospital’.

Figure S1 shows hospital admissions having blood culture specimens collected. Most admissions (379,823 admission from 261,164 patients) had no blood culture specimens collected. 63,374 admissions (from 51,485 patients) had blood culture specimens collected within ±1 calendar days of the day when a parenteral antibiotic was started. The remaining 41,030 admissions (from 36,780 patients) had blood culture specimens collected but not within ±1 calendar day of the day when a parenteral antibiotic was first prescribed.

A sensitivity analysis was conducted by estimating the proportion of patients having a blood culture taken within ±1 calendar day of the day when a parenteral antibiotic was started at the study hospital and continued for at least four consecutive days’ but changing the allowable period of blood culture to (a) within ±2 calendar day and (b) only on the calendar day when parenteral antibiotic was first prescribed. The proportion of patients having blood culture sampled within ±2 calendar days when parenteral antibiotic was first prescribed was 45% (47,455/106,341), and the proportion of patients having blood culture sampled on the calendar day when parenteral antibiotic was first prescribed was 25% (26,167/106,341).

**Supplementary references**

1. Department of Medical Sciences, Ministry of Public Health, Thailand. The laboratory manual: bacteria and fungi for referral and general hospitals. 2015. <http://narst.dmsc.moph.go.th/manuals/Idenbook.pdf>
2. Clinical and Laboratory Standards Institute. Performance Standards for Antimicrobial. Susceptibility Testing. Twentieth Informational Supplement. CLSI document M100-S20, Wayne, PA, 2010
3. Clinical and Laboratory Standards Institute. Performance Standards for Antimicrobial. Susceptibility Testing. Twenty-Fourth Informational Supplement. CLSI document M100-S24, Wayne, PA, 2010

**Table S1.** Estimated proportions and incidence rates of 3^rd^ generation cephalosporin resistant *E. coli* (3GCREC) BSI if 50%, 25% and 10% of the first blood culture episodes were sampled

| **Parameters** | **Based on All data** | **If 50% were sampled^†^** | **If 25% were sampled^†^** | **If 10% were sampled^†^** |
| --- | --- | --- | --- | --- |
| Proportions of 3GCREC | | | | |
| Total | 44% (868/1959) | 48% (446/921) | 52% (320/616) | 57% (224/392) |
| Community-origin | - 42% (631/1514) | 42% (266/629) | 42% (156/368) | 42% (71/174) |
| Hospital-origin | - 53% (237/445) | 61% (179/292) | 66% (163/249) | 70% (153/218) |
| Incidence rates (per 100,000 population per year) | | | | |
| Total | 9.5 (868*10^5^ /9181598) | 4.9 (446*10^5^/9181598) | 3.5 (320*10^5^/9181598) | 2.4 (224*10^5^/9181598) |
| Community-origin | 6.9 (631*10^5^ /9181598) | 2.9 (266*10^5^/9181598) | 1.7 (156*10^5^/9181598) | 0.8 (71*10^5^/9181598) |
| Hospital-origin | 2.6 (237*10^5^ /9181598) | 1.9 (179*10^5^/9181598) | 1.8 (163*10^5^/9181598) | 1.7 (153*10^5^/9181598) |
| Incidence rates (per 100,000 tested patients) | | | | |
| Total | 1,071 (868*10^5^ /81036) | 985 (446*10^5^/45259) | 944 (320*10^5^/33910) | 890 (224*10^5^/25170) |
| Community-origin | 933 (631*10^5^ /67641) | 876 (266*10^5^/30364) | 856 (156*10^5^/18225) | 817 (71*10^5^/8694) |
| Hospital-origin | 1,318 (237*10^5^ /17983) | 1009 (179*10^5^/17732) | 925 (163*10^5^/17614) | 874 (153*10^5^/17504) |
| Blood culture utilization rates (per 1,000 patient-days) | | | | |
|  | 82 (242098*1000/2956643) | 51 (150769*1000/2942386) | 36 (105093*1000/2942386) | 26 (77690*1000/2942386) |
| Proportions of patients having a blood culture taken within ±1 calendar day of the day when a parenteral antibiotic was started and continued for at least four consecutive days. | | | | |
| Total | 44% (47132/106341) | 19% (19523/102422) | 11% (11668/102422) | 6% (5931/102422) |
| Community-origin | 46% (41489/90685) | 16% (16803/90581) | 11% (9790/90581) | 5% (4663/90581) |
| Hospital-origin | 36% (5643/15656) | 23% (2714/11841) | 16% (1880/11841) | 11% (1265/11841) |

**^†^**Median values of 200 bootstrap iterations are presented.

**Table S2.** Estimated proportions and incidence rates of 3^rd^ generation cephalosporin resistant *K. pneumoniae* (3GCRKP) BSI if 50%, 25% and 10% of the first blood culture episodes were sampled

| **Parameters** | **Based on All data** | **If 50% were sampled^†^** | **If 25% were sampled^†^** | **If 10% were sampled^†^** |
| --- | --- | --- | --- | --- |
| Proportions of 3GCRKP | | | | |
| Total | 51% (534/1045) | 61% (368/599) | 68% (318/471) | 75% (284/379) |
| Community-origin | - 34% (193/569) | 35% (80/229) | 35% (46/131) | 34% (21/61) |
| Hospital-origin | - 72% (341/476) | 78% (288/370) | 81% (274/340) | 83% (263/318) |
| Incidence rates (per 100,000 population per year) | | | | |
| Total | 5.8 (534*10^5^ /9181598) | 4.0 (368*10^5^/9181598) | 3.5 (318*10^5^/9181598) | 3.1 (284*10^5^/9181598) |
| Community-origin | 2.1 (193*10^5^ /9181598) | 0.9 (80*10^5^/9181598) | 0.5 (46*10^5^/9181598) | 0.2 (21*10^5^/9181598) |
| Hospital-origin | 3.7 (341*10^5^ /9181598) | 3.1 (288*10^5^/9181598) | 3.0 (274*10^5^/9181598) | 2.9 (263*10^5^/9181598) |
| Incidence rates (per 100,000 tested patients) | | | | |
| Total | 659 (534*10^5^ /81036) | 813 (368*10^5^/45259) | 938 (318*10^5^/33910) | 1128 (284*10^5^/25170) |
| Community-origin | 285 (193*10^5^ /67641) | 262 (80*10^5^/30364) | 252 (46*10^5^/18225) | 242 (21*10^5^/8694) |
| Hospital-origin | 1,896 (341*10^5^ /17983) | 1624 (288*10^5^/17732) | 1556 (274*10^5^/17614) | 1503 (263*10^5^/17504) |

**^†^**Median values of 200 bootstrap iterations are presented.

**Table S3.** Estimated proportions and incidence rates of Methicillin-resistant *S. aureus* (MRSA) BSI if 50%, 25% and 10% of the first blood culture episode were sampled

| **Parameters** | **Based on All data** | **If 50% were sampled^†^** | **If 25% were sampled^†^** | **If 10% were sampled^†^** |
| --- | --- | --- | --- | --- |
| Proportions of MRSA | | | | |
| Total | 23% (252/1092) | 31% (194/623) | 37% (177/483) | 43% (163/380) |
| Community-origin | - 14% (103/750) | 14% (47/325) | 15% (29/196) | 15% (15/101) |
| Hospital-origin | - 44% (149/342) | 49% (147/298) | 52% (148/288) | 53% (148/280) |
| Incidence rates (per 100,000 population per year) | | | | |
| Total | 2.7 (252*10^5^ /9181598) | 2.1 (194*10^5^/9181598) | 1.9 (177*10^5^/9181598) | 1.8 (163*10^5^/9181598) |
| Community-origin | 1.1 (103*10^5^ /9181598) | 0.5 (47*10^5^/9181598) | 0.3 (29*10^5^/9181598) | 0.2 (15*10^5^/9181598) |
| Hospital-origin | 1.6 (149*10^5^ /9181598) | 1.6 (147*10^5^/9181598) | 1.6 (148*10^5^/9181598) | 1.6 (148*10^5^/9181598) |
| Incidence rates (per 100,000 tested patients) | | | | |
| Total | 311 (252*10^5^ /81036) | 429 (194*10^5^/45259) | 522 (177*10^5^/33910) | 648 (163*10^5^/25170) |
| Community-origin | 152 (103*10^5^ /67641) | 155 (47*10^5^/30364) | 159 (29*10^5^/18225) | 173 (15*10^5^/8694) |
| Hospital-origin | 829 (149*10^5^ /17983) | 829 (147*10^5^/17732) | 840 (148*10^5^/17614) | 846 (148*10^5^/17504) |

**^†^**Median values of 200 bootstrap iterations are presented.

**Table S4.** Estimated proportions and incidence rates of carbapenem resistant *Acinetobacter* spp (CRACI) BSI if 50%, 25% and 10% of the first blood culture episodes were sampled

| **Parameters** | **Based on All data** | **If 50% were sampled^†^** | **If 25% were sampled^†^** | **If 10% were sampled^†^** |
| --- | --- | --- | --- | --- |
| Proportions of CRACI | | | | |
| Total | 65% (647/1000) | 74% (547/743) | 77% (520/671) | 81% (497/616) |
| Community-origin | - 33% (80/242) | 34% (33/98) | 34% (20/58) | 32% (9/28) |
| Hospital-origin | - 75% (567/758) | 80% (515/645) | 81% (500/613) | 83% (489/589) |
| Incidence rates (per 100,000 population per year) | | | | |
| Total | 7.0 (647*10^5^ /9181598) | 6.1 (547*10^5^/9181598) | 5.7 (520*10^5^/9181598) | 5.4 (497*10^5^/9181598) |
| Community-origin | 0.9 (80*10^5^ /9181598) | 0.4 (33*10^5^/9181598) | 0.2 (20*10^5^/9181598) | 0.1 (9*10^5^/9181598) |
| Hospital-origin | 6.2 (567*10^5^ /9181598) | 5.6 (515*10^5^/9181598) | 5.4 (500*10^5^/9181598) | 5.3 (489*10^5^/9181598) |
| Incidence rates (per 100,000 tested patients) | | | | |
| Total | 798 (647*10^5^ /81036) | 1209 (547*10^5^/45259) | 1532 (520*10^5^/33910) | 1975 (497*10^5^/25170) |
| Community-origin | 118 (80*10^5^ /67641) | 109 (33*10^5^/30364) | 110 (20*10^5^/18225) | 104 (9*10^5^/8694) |
| Hospital-origin | 3153 (567*10^5^ /17983) | 2904 (515*10^5^/17732) | 2836 (500*10^5^/17614) | 2794 (489*10^5^/17504) |

**^†^**Median values of 200 bootstrap iterations are presented

**Table S5.** Estimated proportions and incidence rates of carbapenem resistant *Pseudomonas aeruginosa* (CRPA) BSI if 50%, 25% and 10% of the first blood culture episodes were sampled

| **Parameters** | **Based on All data** | **If 50% were sampled^†^** | **If 25% were sampled^†^** | **If 10% were sampled^†^** |
| --- | --- | --- | --- | --- |
| Proportions of CRPA | | | | |
| Total | 28% (125/450) | 35% (101/291) | 39% (95/246) | 43% (90/211) |
| Community-origin | - 14% (23/170) | 13% (9/69) | 13% (5/40) | 12% (2/17) |
| Hospital-origin | - 36% (102/280) | 41% (92/222) | 43% (90/206) | 45% (87/194) |
| Incidence rates (per 100,000 population per year) | | | | |
| Total | 1.4 (125*10^5^ /9181598) | 1.1 (101*10^5^/9181598) | 1.0 (95*10^5^/9181598) | 1.0 (90*10^5^/9181598) |
| Community-origin | 0.3 (23*10^5^ /9181598) | 0.1 (9*10^5^/9181598) | 0.1 (5*10^5^/9181598) | 0.02 (2*10^5^/9181598) |
| Hospital-origin | 1.1 (102*10^5^ /9181598) | 1.0 (92*10^5^/9181598) | 1.0 (90*10^5^/9181598) | 0.9 (87*10^5^/9181598) |
| Incidence rates (per 100,000 tested patients) | | | | |
| Total | 154 (125*10^5^ /81036) | 223 (101*10^5^/45259) | 280 (95*10^5^/33910) | 358 (90*10^5^/25170) |
| Community-origin | 34 (23*10^5^ /67641) | 30 (9*10^5^/30364) | 27 (5*10^5^/18225) | 23 (2*10^5^/8694) |
| Hospital-origin | 567 (102*10^5^ /17983) | 519 (92*10^5^/17732) | 508 (90*10^5^/17614) | 497 (87*10^5^/17504) |

**^†^**Median values of 200 bootstrap iterations are presented

**Table S6.** Characteristics of patients with BSI of hospital-origin caused by *E. coli* (N=445) stratified by exposure to a parenteral antibiotic at the study hospital

| Characteristics | First positive blood culture was sampled within ±1 calendar day when a parenteral antibiotic was started and continued for at least four consecutive days**^†^** | First positive blood culture was sampled outside ±1 calendar day when a parenteral antibiotic was started and continued for at least four consecutive days**^†^** | First positive blood culture was sampled at any time point when a parenteral antibiotic was given less than four consecutive days**^††^** | P value |
| --- | --- | --- | --- | --- |
| Proportions of 3GCREC (%) | 30% (63/207) | 79% (166/210) | 29% (8/28) | <0.001 |
| Time from hospital admission to the collection of the first blood culture specimen positive for *E. coli*  (calendar days; median, IQR, N) | 5 (3-12, N=207) | 10.5 (5-23, N=210) | 4 (3-5, N=28) | <0.001 |
| Time from the start of a parenteral antibiotic to the collection of the first blood culture specimen positive for *E. coli*  (calendar days; median, IQR, N) | 0 (0-1, N=207) | 7.5 (4-21, N=210) | 0 (0-1, N=24)**^†††^** | <0.001 |
| In-hospital mortality (%) | 18% (37/207) | 26% (55/210) | 4% (1/28) | 0.006 |

3GCREC = 3rd generation cephalosporin resistant *E. coli*; IQR = Interquartile range (25^th^ and 75^th^ percentile)

**^†^**Included patients who died, were discharged to a hospice or transferred to another hospital before completing four consecutive days of antibiotics and had antibiotics continuously until the day prior to death, hospice discharge or transfer, respectively

**^††^**Excluded patients who died, were discharged to a hospice or transferred to another hospital before completing four consecutive days of antibiotics and had antibiotics continuously until the day prior to death, hospice discharge or transfer, respectively

**^†††^**Four patients received no parenteral antibiotics

**Table S7.** Characteristics of patients with BSI of hospital-origin caused by *K. pneumoniae* (N=476) stratified by exposure to a parenteral antibiotic at the study hospital

| Characteristics | First positive blood culture was sampled within ±1 calendar day when a parenteral antibiotic was started and continued for at least four consecutive days**^†^** | First positive blood culture was sampled outside ±1 calendar day when a parenteral antibiotic was started and continued for at least four consecutive days**^†^** | First positive blood culture was sampled at any time point when a parenteral antibiotic was given less than four consecutive days**^††^** | P value |
| --- | --- | --- | --- | --- |
| Proportions of 3GCNS *K. pneumoniae* (%) | 37% (45/122) | 86% (292/341) | 31% (4/13) | <0.001 |
| Time from hospital admission to the collection of the first blood culture specimen positive for *K. pneumoniae*  (calendar days; median, IQR, N) | 6.5 (4-12; N=122) | 14 (8-27; N=341) | 6 (4-7; N=13) | <0.001 |
| Time from the start of parenteral antibiotic to the collection of the first blood culture specimen positive for *K. pneumoniae* (calendar days; median, IQR, N) | 0 (0-1; N=122) | 11 (6-23; N=341) | 0 (0-0; N=9)**^†††^** | <0.001 |
| In-hospital mortality (%) | 28% (34/122) | 35% (118/341) | 0% (0/13) | 0.009 |

3GCRKP = 3rd generation cephalosporin resistant *K. pneumoniae*; IQR = Interquartile range (25^th^ and 75^th^ percentile)

**^†^**Included patients who died, were discharged to a hospice or transferred to another hospital before completing four consecutive days of antibiotics and had antibiotics continuously until the day prior to death, hospice discharge or transfer, respectively

**^††^**Excluded patients who died, were discharged to a hospice or transferred to another hospital before completing four consecutive days of antibiotics and had antibiotics continuously until the day prior to death, hospice discharge or transfer, respectively

**^†††^**Four patients received no parenteral antibiotics

**Table S8.** Characteristics of patients with BSI of hospital-origin caused by *S. aureus* (N=342) stratified by exposure to a parenteral antibiotic at the study hospital

| Characteristics | First positive blood culture was sampled within ±1 calendar day when a parenteral antibiotic was started and continued for at least four consecutive days**^†^** | First positive blood culture was sampled outside ±1 calendar day when a parenteral antibiotic was started and continued for at least four consecutive days**^†^** | First positive blood culture was sampled at any time point when a parenteral antibiotic was given less than four consecutive days**^††^** | P value |
| --- | --- | --- | --- | --- |
| Proportions of MRSA (%) | 9% (12/130) | 74% (135/183) | 7% (2/29) | <0.001 |
| Time from hospital admission to the collection of the first blood culture specimen positive for *S. aureus*  (calendar days; median, IQR, N) | 5 (3-9, N=130) | 14 (8-27; N=183) | 4 (3-6, N=29) | <0.001 |
| Time from the start of parenteral antibiotic to the collection of the first blood culture specimen positive for *S. aureus*  (calendar days; median, IQR, N) | 0 (0-0, N=130) | 12 (6-25, N=183) | 0 (0-0, N=18)**^†††^** | <0.001 |
| In-hospital mortality (%) | 22% (28/130) | 34% (62/183) | 7% (2/29) | <0.001 |

MRSA = Methicillin-resistant *S. aureus*; IQR = Interquartile range (25^th^ and 75^th^ percentile)

**^†^**Included patients who died, were discharged to a hospice or transferred to another hospital before completing four consecutive days of antibiotics and had antibiotics continuously until the day prior to death, hospice discharge or transfer, respectively

**^††^**Excluded patients who died, were discharged to a hospice or transferred to another hospital before completing four consecutive days of antibiotics and had antibiotics continuously until the day prior to death, hospice discharge or transfer, respectively

**^†††^**Eleven patients received no parenteral antibiotics

**Table S9.** Characteristics of patients with BSI of hospital-origin caused by *Acinetobacter* spp (N=758) stratified by exposure to a parenteral antibiotic at the study hospital

| Characteristics | First positive blood culture was taken within ±1 calendar day when a parenteral antibiotic was started and continued for at least four consecutive days**^†^** | First positive blood culture was taken outside ±1 calendar day when a parenteral antibiotic was started and continued for at least four consecutive days**^†^** | First positive blood culture was taken at any time point when a parenteral antibiotic was given less than four consecutive days**^††^** | P value |
| --- | --- | --- | --- | --- |
| Proportions of CRACI (%) | 32% (25/78) | 81% (540/663) | 12% (2/17) | <0.001 |
| Time from hospital admission to the collection of the first blood culture specimen positive for *Acinetobacter* spp  (calendar days; median, IQR, N) | 6 (4-8, N=78) | 11 (7-19, N=663) | 4 (3-5, N=17) | <0.001 |
| Time from the start of a parenteral antibiotic to the collection of the first blood culture specimen positive for *Acinetobacter* spp  (calendar days; median, IQR, N) | 0 (0-1, N=78) | 9 (5-16, N=663) | 0 (0-0, N=6)**^†††^** | <0.001 |
| In-hospital mortality (%) | 23% (18/78) | 44% (294/663) | 0% (0/17) | <0.001 |

CRACI = carbapenem resistant *Acinetobacter* spp; IQR = Interquartile range (25^th^ and 75^th^ percentile)

**^†^**Included patients who died, were discharged to a hospice or transferred to another hospital before completing four consecutive days of antibiotics and had antibiotics continuously until the day prior to death, hospice discharge or transfer, respectively

**^††^**Excluded patients who died, were discharged to a hospice or transferred to another hospital before completing four consecutive days of antibiotics and had antibiotics continuously until the day prior to death, hospice discharge or transfer, respectively

**^†††^**Eleven patients received no parenteral antibiotics

**Table S10.** Characteristics of patients with BSI of hospital-origin caused by *P. aeruginosa* (N=758) stratified by exposure to a parenteral antibiotic at the study hospital

| Characteristics | First positive blood culture was taken within ±1 calendar day when a parenteral antibiotic was started and continued for at least four consecutive days**^†^** | First positive blood culture was taken outside ±1 calendar day when a parenteral antibiotic was started and continued for at least four consecutive days**^†^** | First positive blood culture was taken at any time point when a parenteral antibiotic was given less than four consecutive days**^††^** | P value |
| --- | --- | --- | --- | --- |
| Proportions of CRPA (%) | 12% (5/42) | 42% (97/231) | 0% (0/7) | <0.001 |
| Time from hospital admission to the collection of the first blood culture specimen positive for *P. aeruginosa*  (calendar days; median, IQR, N) | 8.5 (5-13, N=42) | 17 (9-30, N=231) | 5 (3-19, N=7) | <0.001 |
| Time from the start of a parenteral antibiotic to the collection of the first blood culture specimen positive for *P. aeruginosa*  (calendar days; median, IQR, N) | 0 (0-0, N=42) | 14 (6-28, N=231) | 0.5 (0-1.5, N=4)**^†††^** | <0.001 |
| In-hospital mortality (%) | 29% (12/42) | 35% (82/231) | 14% (1/7) | 0.45 |

CRPA = carbapenem resistant *P. aeruginosa*; IQR = Interquartile range (25^th^ and 75^th^ percentile)

**^†^**Included patients who died, were discharged to a hospice or transferred to another hospital before completing four consecutive days of antibiotics and had antibiotics continuously until the day prior to death, hospice discharge or transfer, respectively

**^††^**Excluded patients who died, were discharged to a hospice or transferred to another hospital before completing four consecutive days of antibiotics and had antibiotics continuously until the day prior to death, hospice discharge or transfer, respectively

**^†††^**Three patients received no parenteral antibiotics

**Figure S1.** Sankey diagram showing hospital admissions having blood culture specimens collected.


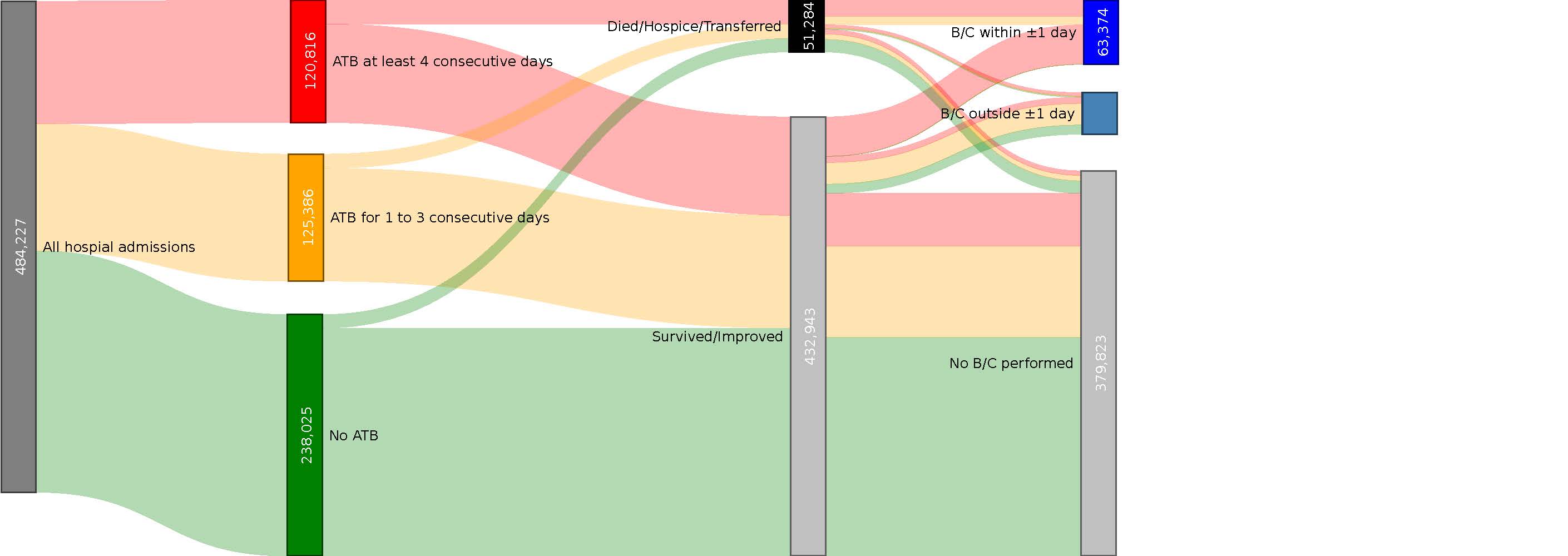


ATB = parenteral antibiotics; B/C = blood culture. B/C within ±1 calendar day is defined as having a blood culture specimen taken within ±1 calendar day of the day when a parenteral antibiotic was started and continued for at least four consecutive days. This group also includes patients who died, were discharged to a hospice or transferred to another hospital within four days after a parenteral antibiotic was started, and a parenteral antibiotic was continued until the day prior to death, hospice discharge or transfer. B/C outside ±1 calendar day is defined as having a blood culture specimen taken outside ±1 calendar day of the day when a parenteral antibiotic was started and continued for at least four consecutive days. This group also includes patients who had no parenteral antibiotic prescribed.

**Figure S2.** Estimated proportions and incidence rates of 3rd generation cephalosporin resistant *K. pneumoniae* (3GCRKP) if 50%, 25%, and 10% of the first blood culture episodes were sampled


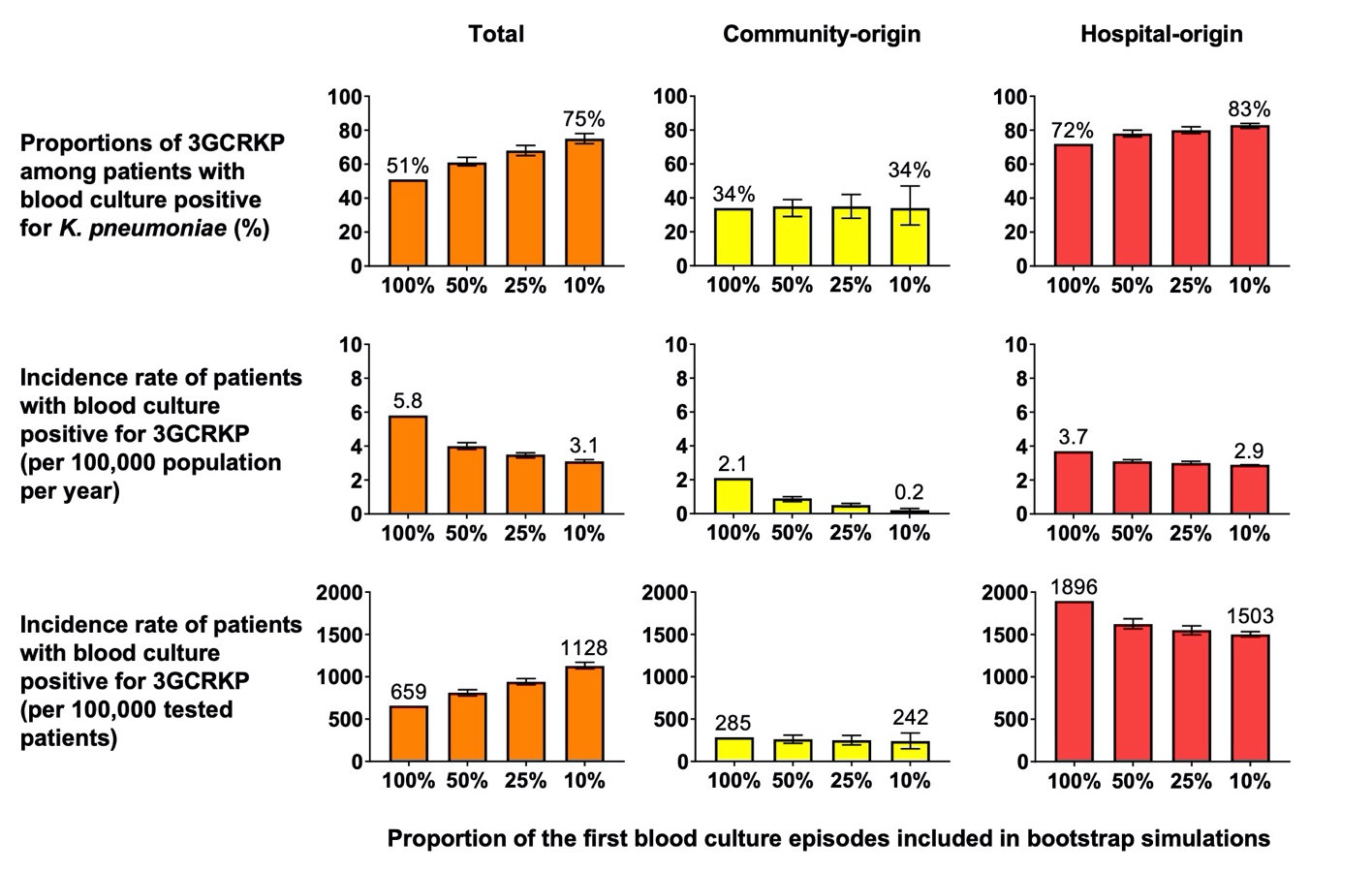


**Figure S3.** Estimated proportions and incidence rates of Methicillin-resistant *S. aureus* (MRSA) if 50%, 25%, and 10% of the first blood culture episodes were sampled

**Figure S4.** Estimated proportions and incidence rates of Carbapenem-resistant *Acinetobacter* spp. (CRACI) if 50%, 25%, and 10% of the first blood culture episodes were sampled

**Figure S5.** Estimated proportions and incidence rates of Carbapenem-resistant *Pseudomonas aeruginosa* (CRPA) if 50%, 25%, and 10% of the first blood culture episodes were sampled
